# Robust Image Encryption with 2D Hyperchaotic Map and Dynamic DNA-Zigzag Encoding

**DOI:** 10.3390/e27060606

**Published:** 2025-06-06

**Authors:** Haijun Zhang, Xiaojiao Liu, Kehan Chen, Rigen Te, Fei Yan

**Affiliations:** 1 Chongqing Research Institute, Changchun University of Science and Technology, Chongqing 401135, China; 2022101109@mails.cust.edu.cn; 2 School of Computer Science and Technology, Changchun University of Science and Technology, Changchun 130022, China; 2022101968@mails.cust.edu.cn (X.L.); chenkehan@cust.edu.cn (K.C.); 3 Chang Guang Satellite Technology Co., Ltd., Changchun 130000, China

**Keywords:** hyperchaotic map, image encryption, shuffle algorithm, DNA-zigzag encoding

## Abstract

This study presents a novel two-dimensional hyperchaotic map, referred to as the 2D exponent-logarithm-sine chaotic map (2D-ELSCM), which is intricately designed through the interplay of exponential, logarithmic, and sine functions. To comprehensively evaluate the chaotic performance of the 2D-ELSCM, several critical metrics are employed, including the largest Lyapunov exponent (LLE), permutation entropy (PE), sample entropy (SE), Kolmogorov entropy (KE), and the results of the 0–1 test, which yield values of 8.3175, 0.9998, 1.9826, 2.1117, and 0.9970, respectively. Furthermore, the 2D-ELSCM successfully passes the NIST randomness tests, collectively confirming its exceptional randomness and complexity. Building upon this robust chaotic map, we develop a distinctive chaotic image encryption scheme that employs an improved Knuth-Durstenfeld shuffle (IKDS) to rearrange pixel positions, effectively disrupting the correlation between adjacent pixels. Complementing this, we introduce a dynamic diffusion mechanism that integrates DNA encoding with the Zigzag transform, thereby promoting global pixel diffusion and enhancing encryption security. The initial conditions of the chaotic map are generated from the SHA-512 hash of the plaintext image in conjunction with an external key, which not only expands the key space but also significantly improves key sensitivity. Simulation results demonstrate that the proposed encryption scheme achieves correlation coefficients approaching 0 in the encrypted test images, with an average NPCR of 99.6090% and UACI of 33.4707%. These findings indicate a strong resistance to various attacks and showcase excellent encryption quality, thereby underscoring the scheme’s potential for secure image transmission and storage.

## 1. Introduction

In an era of rapid technological advancement and exponential growth in digital data, ensuring the security of multimedia information has become a critical concern. Among various forms of multimedia, images are particularly significant in applications such as medical imaging, military reconnaissance, and secure communication. However, the widespread distribution and exchange of digital images over public networks expose them to risks such as unauthorized access, tampering, and data theft. As a result, the development of robust image encryption techniques has become a key area of research in information security. Traditional encryption algorithms, such as the Advanced Encryption Standard (AES) [1] and the Rivest-Shamir-Adleman (RSA) algorithm [2], have been widely used for securing textual data. While these methods are effective for general-purpose encryption, they often struggle with the large data volumes and pixel-level correlations inherent in digital images. These limitations have driven researchers to explore novel approaches specifically designed for image encryption, with the goal of achieving high security, efficiency, and robustness against various cryptographic attacks.

In recent years, chaos theory has garnered significant attention in image encryption due to its unique characteristics, such as sensitivity to initial conditions, ergodicity, and unpredictability. Chaotic systems used in encryption are typically categorized into one-dimensional (1D) and high-dimensional (HD) types. 1D chaotic systems are widely adopted in chaos-based image encryption algorithms because of their simplicity in implementation. For instance, Zhou et al. [3] proposed a novel combination chaotic system (NCCS) for image encryption, demonstrating strong chaotic behavior and robust security. Liang et al. [4] introduced a 1D Sine-Cosine chaotic map (SCCM) and developed an image encryption algorithm that integrates SCCM with random DNA operations. Similarly, Kocak et al. [5] introduced a 1D modular integrated logistic exponential (MILE) map and applied it to a PSO-based image encryption scheme. Despite their effectiveness, image encryption algorithms based on 1D chaotic systems are typically constrained by narrow parameter ranges and weak sensitivity, which limits their ability to provide robust information security. As a result, researchers have increasingly focused on HD chaotic systems, which provide larger key spaces and more complex chaotic behavior, thereby helping to address these limitations [6]. Wang et al. [7] proposed a method utilizing a four-dimensional (4D) chaotic system to generate four chaotic sequences, which were then employed to determine the DNA encoding, decoding, and operational methods. Darani et al. [8] introduced a secure cryptographic algorithm based on a novel three-dimensional (3D) chaotic system (P3DCS), which effectively resists cropping and noise attacks. However, HD chaotic systems tend to be more expensive to implement in terms of both hardware and software due to their higher computational complexity. Therefore, finding the right balance between 1D and HD chaotic maps to design an optimal chaotic map has become a significant challenge. In this context, two-dimensional (2D) chaotic systems are often preferred due to their ease of implementation and strong randomness, making them widely adopted. For example, Wang et al. [9] developed an image encryption algorithm based on a new 2D chaotic system, 2D-SLTCM, achieving high-security performance. Chen et al. [10] proposed a novel 2D logarithm-exponent-squared-sine coupling map (2D-LESSCM), and designed an adaptive image compression and encryption scheme, demonstrating exceptional security and robustness. Pak et al. [11] proposed a novel 2D Infinite Collapse Coupling Map (2D-ICCM) and designed a simple-structured pixel-level image encryption algorithm based on 2D-ICCM to achieve higher security and efficiency. More recently, Tang et al. [12] proposed a 2D sine-power coupling map (2D-SPCM) exhibiting enhanced chaotic behavior, and developed a cross-plane color image encryption algorithm (CPCIE-SPCM) integrated within an asymmetric encryption framework (ACIES-ECC), further improving key security and resistance to ciphertext tampering. Du et al. [13] constructed a cross-feedback discrete hyperchaotic system (LCF-CFDHS) by coupling one 2D and two 1D chaotic maps, significantly expanding the chaotic region and improving encryption robustness. Zhou et al. [14] introduced a novel 2D hyperchaotic map with larger Lyapunov exponents and designed a dynamic RNA computing-based encryption method with enhanced security.

Although 2D chaotic systems are widely used in image encryption due to their simplicity and strong randomness, they still face several limitations. Many existing 2D chaotic maps suffer from small key spaces, low complexity, and periodicity issues, making them susceptible to brute-force and statistical attacks. Additionally, their randomness and sensitivity may not fully meet modern security demands. To address these issues, this study proposes a novel 2D hyperchaotic map, the 2D exponent-logarithm-sine chaotic map (2D-ELSCM), which integrates exponential, logarithmic, and sine functions to provide a larger key space and enhanced unpredictability. Performance evaluations demonstrate its potential for robust image encryption.

However, achieving an optimal balance between security and computational efficiency remains a significant challenge in the field of image encryption. To address this, researchers have explored hybrid approaches that combine chaotic maps with complementary techniques. Among these, DNA-based encryption has emerged as a promising solution due to its high information density and parallel processing capabilities [15]. By leveraging the molecular structure of DNA, researchers have proposed encryption schemes that encode digital information into DNA sequences, offering a novel biologically inspired approach to ensure secure data transmission. For instance, Yu et al. [16] proposed an image encryption algorithm that integrates a novel DNA sequence operation with a hyperchaotic system, introducing a DNA triploid mutation (DNA-TM) operation to achieve cryptographic transformation of DNA bases. Similarly, Wang et al. [17] designed a 2D hyperchaotic map (2D-CICM), which combines the Cubic map with the Infinite Collapse Map (ICM) and incorporates a 3D L-shaped transformation along with DNA crossover and mutation techniques to further enhance security. Additionally, Kumar et al. [18] utilized an innovative exponent-sine-cosine (ESC) chaotic map in conjunction with dynamic permutation and DNA-based diffusion to achieve multiple-image encryption. Furthermore, Zhou et al. [19] developed a multi-image encryption scheme based on a new 2D hyperchaotic model combined with cyclic shift coding of DNA. By introducing a cross-coupling chaotic system and a DNA cyclic shift encoding strategy, their approach not only reduced system complexity but also improved the randomness, security, and resistance against differential attacks.

Although DNA-based encryption techniques have made significant progress, numerous challenges still remain. Many existing schemes rely solely on a limited set of DNA rules, which restricts the key space and makes them vulnerable to brute-force attacks. Moreover, the inadequate integration with chaotic systems may result in insufficient randomness and reduced sensitivity to initial conditions, thereby weakening the overall encryption strength. To address these challenges, it is essential to optimize the DNA encoding process, improve key generation mechanisms, and incorporate advanced chaotic systems to enhance encryption randomness. Based on these considerations, this paper proposes an image encryption algorithm that integrates chaotic systems with an improved DNA diffusion technique to enhance encryption performance. The contributions of this study are as follows: (i) The 2D-ELSCM is designed to provide a larger key space and more complex chaotic dynamics, increasing randomness and security. (ii) The improved DNA encoding and Zigzag transformation are integrated to achieve efficient pixel diffusion, thereby enhancing attack resistance and encryption strength. (iii) An optimized key generation mechanism and an efficient image encryption scheme are proposed to balance security and computational efficiency, meeting practical application requirements.

The rest of this paper is structured as follows: Section 2 provides an overview of the techniques employed in this study. Section 3 introduces the proposed 2D-ELSCM hyperchaotic system and highlights its superior performance. Section 4 elaborates on the proposed encryption algorithm, including its architecture, key generation process, and encryption procedures. Section 5 presents the experimental results and security analysis. Finally, Section 6 summarizes the paper and suggests potential future research directions.

## 2. Preliminary Works

### 2.1. Classic Knuth-Durstenfeld Shuffle Algorithm

The classic Knuth-Durstenfeld shuffle (CKDS) algorithm [20], an optimized version of the Fisher-Yates shuffle, is a widely adopted method for generating unbiased random permutations of a finite sequence. It operates in-place with a time complexity of O(n), making it both computationally efficient and memory-conserving.

The algorithm operates by iterating backward through the array, starting from the last element. At each step, it randomly selects an index from the unshuffled portion of the array and swaps the corresponding elements. This process guarantees that all possible permutations are equally probable. The pseudocode for this algorithm is presented in Algorithm 1. Owing to its efficiency and reliability, CKDS algorithm finds extensive applications in cryptographic systems, random sampling, and other scenarios requiring unbiased data shuffling.
**Algorithm 1** Pseudocode for CKDS algorithm**Require:** 
Array arr of size *n*; temporary variable temp;1:n←length(arr)2:**for** i=n−1 to 1 **do**3: # Generate random index from 0 to *i*4: j←RandomInteger(0,i)5: temp←arr[i]6: arr[i]←arr[j]7: arr[j]←temp8:**end for**9:**return** arr

### 2.2. Zigzag Transformation

Zigzag transformation [21] is a widely employed technique in image processing and encryption, designed to enhancing the security and randomness of data arrangements. The primary function of the Zigzag operation is to reorder the elements of a matrix in a non-linear manner, following a predefined diagonal traversal pattern.

**Figure 1:**
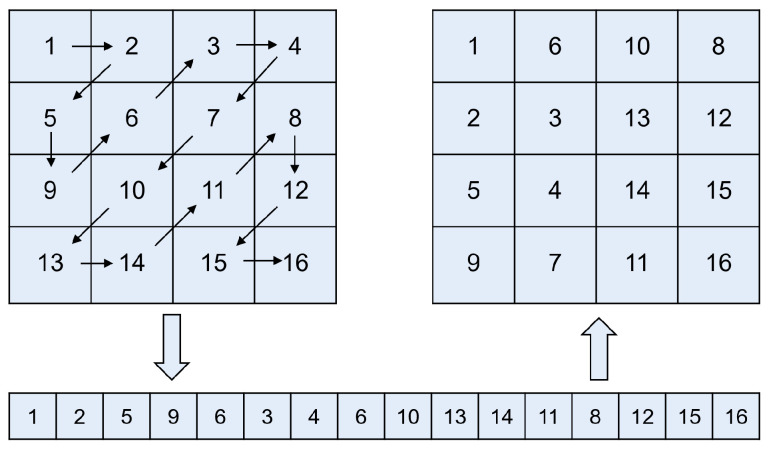
Scanning process of the standard Zigzag transformation.

Typically, the Zigzag operation begins at the top-left corner of the matrix and moves diagonally, alternating between upward and downward directions, until all elements are traversed. This traversal pattern transforms the original sequential arrangement of data into a scrambled order, effectively reducing spatial correlation among neighboring elements. Figure 1 illustrates the scanning process in detail for a fourth-order square matrix.

### 2.3. DNA Coding

A DNA sequence is composed of four nucleotides [4]: A (adenine), T (thymine), C (cytosine), and G (guanine), where A pairs with T and C pairs with G based on the principle of complementary base pairing. Similarly, in binary sequences, 0 and 1 are considered complements. Based on this analogy, the nucleotides A, T, C, and G can be represented by binary values such as 00, 01, 10, and 11, respectively. This mapping enables the four nucleotides to correspond to four unique binary codes, resulting in a total of 24 possible encoding schemes. However, only 8 of these encoding methods comply with the Watson-Crick complementarity rule [22], as illustrated in Table 1.

For instance, the pixel value 45 of the image can be expressed in binary as “00101101”, and by applying coding rule 3 from Table 1, the DNA sequence “TGAC” can be derived. Likewise, decoding the DNA sequence “TGAC” using rule 3 reconstructs the binary value “00101101” corresponding to the pixel value 45. However, using other rules for decoding results in a different value. For example, applying rule 8 to the binary number “01001011” yields the pixel value 180. It is evident that the DNA encoding and decoding processes effectively alter the pixel values, thereby providing an encryption effect. For DNA sequences, operations such as addition, subtraction, XOR, and complementary transformations are commonly employed to enhance the complexity of the sequence. Table 2 provides a detailed demonstration of the XOR operation applied to nucleic acid bases, illustrating how this process contributes to the overall robustness of DNA-based encryption schemes.

## 3. Hyperchaotic Map

In this section, we present the mathematical definition of the 2D-ELSCM and assess its performance based on specific assessment indicators. Furthermore, the proposed hyperchaotic map demonstrates superior behavior compared to several existing chaotic systems in terms of quantitative evaluation metrics. The results suggest that the dynamical characteristics of this system surpass those of other systems, exhibiting higher complexity and enhanced randomness.

### 3.1. Definition of 2D-ELSCM

To address the limitations of traditional 1D and HD chaotic systems, this study proposes a novel 2D chaotic model that couples a logarithmic-exponential function with a squared sine function, as defined in Equation (1):

(1) $$\left\{\begin{array}{c} x_{i+1}=sin^{2}\left[m\pi^{2}\left(ln\left(x_{i}\right)exp\left(y_{i}\right)+ln\left(y_{i}\right)exp\left(x_{i}\right)\right)\right]\\ y_{i+1}=sin^{2}\left[n\pi^{2}\left(ln\left(x_{i}y_{i}\right)exp\left(x_{i}y_{i}\right)\right)\right] \end{array}\right.$$

where $x_{i}$ and $y_{i}$ represent the states of 2D-ELSCM, and *m* and *n* are control parameters with m,n≠0.

The proposed system exhibits strong nonlinear dynamics due to the combined effects of the logarithmic function ln(·) and the exponential function exp(·). The singular behavior of the logarithmic function, coupled with the rapid growth of the exponential function, enhances the chaotic characteristics of the system, particularly as the values of $x_{i}$ and $y_{i}$ vary, leading to high sensitivity to initial conditions. Additionally, the use of the $sin^{2}\left(\cdot \right)$ function constrains the iterative results to the range [0,1]. This newly proposed map not only amplifies the unpredictability of numerical values but also further enhances the chaotic nature of the system, making it highly suitable for applications requiring robust chaotic behavior.

### 3.2. Performance of 2D-ELSCM

This section analyzes the 2D-ELSCM using various test metrics, including bifurcation diagrams, trajectory diagrams, Lyapunov exponent, sample entropy, permutation entropy, Kolmogorov entropy, the 0–1 test, and the NIST randomness test. Furthermore, the newly proposed map is compared with six existing 2D chaotic maps to provide a more comprehensive performance analysis. The mathematical definitions of these maps are concisely summarized in Table 3.

#### 3.2.1. Bifurcation and Phase Plane Trajectory Diagram

The bifurcation diagram highlights the elements of the chaotic sequence with respect to control parameters [28], providing a direct visualization of the system’s evolution process.

**Figure 2:**
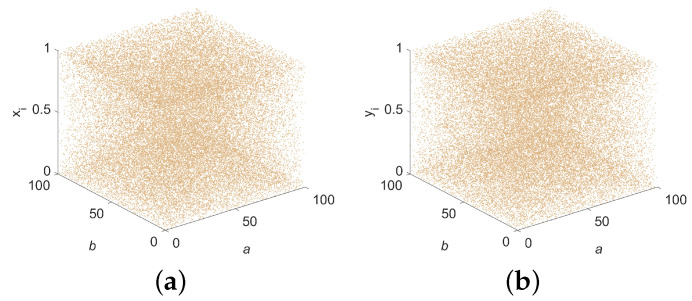
Bifurcation diagram of 2D-ELSCM. (**a**) State variable $x_{i}$; (**b**) State variable $y_{i}$.

In this section, the bifurcation diagram of the 2D-ELSCM is presented in Figure 2, where the initial values ($x_{0},y_{0}$) are set to (0.5, 0.5). Figure 2a illustrates the variation of the state variable $x_{i}$ as the control parameters a∈(0,100] and b∈(0,100] change. Similarly, Figure 2b demonstrates the variation of the state variable $y_{i}$ under the same conditions.

The 3D bifurcation diagram of the 2D-ELSCM, also shown in Figure 2, depicts how the state variables $x_{i}$ and $y_{i}$ evolve as the control parameters vary over the same range. Clearly, the proposed system spans the entire parameter space rather than being confined to specific regions. This indicates that the hyperchaotic sequences generated by the system exhibit high sensitivity to initial conditions and control parameters, as well as strong ergodicity.

**Figure 3:**
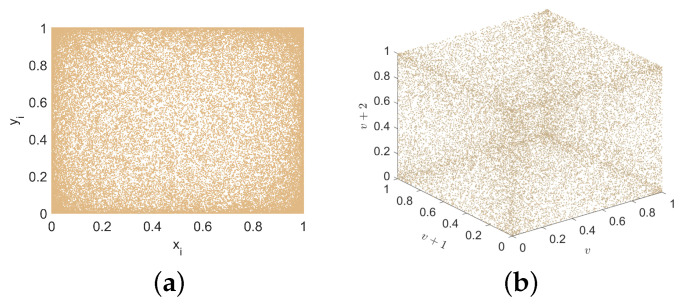
Trajectory diagram of 2D-ELSCM for *a* = 15 and *b* = 15 (Initial values $x_{0}$ = 0.5, $y_{0}$ = 0.5). (**a**) 2D trajectory; (**b**) 3D trajectory.

The phase space trajectory provides an intuitive method for observing system dynamics. When the points representing the chaotic sequence densely populate the coordinate space, it implies that the nonlinear system’s output exhibits enhanced randomness. Figure 3a presents 2D phase space trajectory diagrams of $x_{i}$ versus $y_{i}$, while Figure 3b illustrates the 3D phase space trajectory formed by *v* + 2, *v* + 1, and *v*, where *v* denotes the combined *x* and *y* series.

Notably, neither Figure 2 nor Figure 3 reveals any evidence of aggregation, underscoring that the proposed 2D hyperchaotic map delivers strong security characteristics, making it well-suited for communication applications.

#### 3.2.2. Lyapunov Exponent

The Lyapunov exponent (LE) serves as an objective metric for evaluating chaotic behavior in nonlinear systems, where the number of exponents corresponds to the dimensionality. A positive LE indicates instability and sensitivity to initial conditions, reflecting inherent unpredictability.

**Figure 4:**
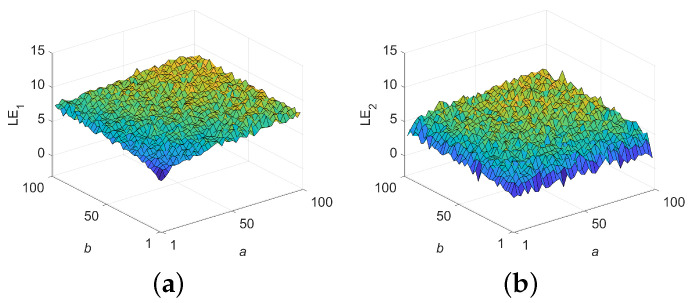
LEs of 2D-ELSCM. (**a**) 3D illustration of $LE_{1}$; (**b**) 3D illustration of $LE_{2}$.

A 2D chaotic system typically has two LEs, denoted as $LE_{1}$ and $LE_{2}$. In this section, the 3D plots of $LE_{1}$ and $LE_{2}$ for 2D-ELSCM, varying with respect to parameters *a* and *b* in the range of [1, 100], are presented in Figure 4. As observed, with changes in the control parameters *a* and *b*, both $LE_{1}$ and $LE_{2}$ remain positive. This confirms that the 2D-ELSCM exhibits hyperchaotic behavior over a broad range.

**Figure 5:**
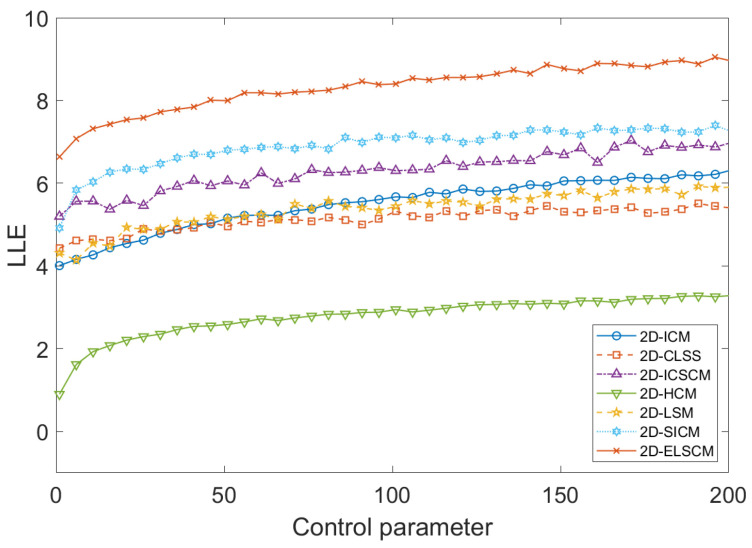
Comparison of LLE of existing 2D maps.

The largest Lyapunov exponent (LLE) is a critical metric for quantifying the chaotic behavior of a dynamic system. A higher LLE value indicates a greater degree of chaos, reflecting stronger sensitivity to initial conditions, as well as increased uncertainty and unpredictability [27]. Figure 5 presents a comparison of the LLE values across different 2D chaotic systems. Additionally, the average LLE values for 2D-ELSCM and other chaotic systems are summarized in Table 4. Notably, the proposed map exhibits larger LLE values, confirming that the 2D-ELSCM demonstrates enhanced dynamic behavior and stronger chaotic properties.

#### 3.2.3. Permutation Entropy

Permutation entropy (PE) is a widely employed metric for quantifying the complexity and chaotic characteristics of a time series, providing valuable insights into the system’s dynamic behavior. By examining the relative ordering patterns of elements within the time series, PE effectively measures the system’s unpredictability and degree of chaos. The PE value ranges from 0 to 1, where values closer to 1 indicate higher complexity and greater chaotic behavior, reflecting highly unpredictable dynamics. Consequently, PE serves as an effective tool for detecting chaotic behavior and evaluating the intrinsic complexity of nonlinear systems.

**Figure 6:**
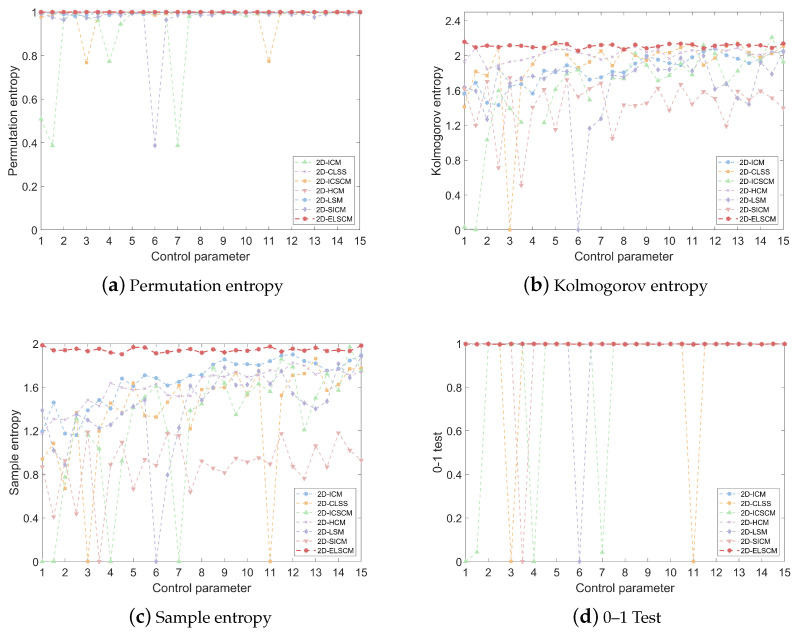
Comparative visualization of chaotic metrics for various 2D chaotic maps.

In the context of the proposed 2D-ELSCM system, a comparative analysis of PE values with several recent chaotic systems is presented in Figure 6a. The average PE values for each system are summarized in Table 4, offering a clear comparison of their chaotic characteristics. From this analysis, it is evident that the 2D-ELSCM achieves the highest mean PE value, outperforming other established chaotic systems. This superior PE value underscores the exceptional randomness and complexity of the 2D-ELSCM, reinforcing its suitability for cryptographic applications where high unpredictability is critical for security.

#### 3.2.4. Kolmogorov Entropy

The Kolmogorov entropy (KE) is a key metric used to assess the randomness or disorder within a dynamical system. It quantifies the rate at which the system’s trajectories diverge, offering insights into the system’s unpredictability. A system with high KE values indicates highly chaotic and irregular behavior, making future states difficult to predict. In contrast, a system with low KE values tends to exhibit more regular or predictable patterns, implying lower complexity and reduced sensitivity to initial conditions.

For the 2D-ELSCM, the KE values are computed using the method proposed by [29]. The KE values of the 2D-ELSCM are then compared with those of six other recently proposed chaotic systems, as shown in Figure 6b. The analysis reveals that the 2D-ELSCM consistently exhibits larger positive KE values across a wide range of parameter settings, outperforming other chaotic maps. This suggests that the proposed system encompasses a broader chaotic range and demonstrates stronger unpredictability. Such characteristics are crucial for cryptographic applications, where a high level of randomness and disorder is necessary to ensure data security.

Furthermore, the average KE values summarized in Table 4 further underscore the superior performance of the 2D-ELSCM. The table illustrates that the proposed map generates chaotic sequences with the highest complexity among those analyzed. This demonstrates that the 2D-ELSCM is not only more chaotic but also capable of offering stronger resistance to attacks, thereby enhancing its effectiveness in cryptographic systems.

#### 3.2.5. Sample Entropy

Sample entropy (SE) is a quantitative measure used to assess the complexity, irregularity, or unpredictability of time series data, particularly in the context of chaotic systems. It improves upon approximate entropy (AE) by focusing on evaluating the likelihood that similar patterns will recur at different times within the series. Essentially, SE quantifies the degree of unpredictability by analyzing the probability of recurrence of similar sequences over time. A higher SE value indicates that the system is more complex, less predictable, and demonstrates a higher level of chaotic behavior, making it a valuable tool for analyzing chaotic systems, especially in cryptographic applications.

In Figure 6c, the SE values for the 2D-ELSCM are presented alongside those from other recently proposed chaotic systems. This comparison underscores the superior performance of the 2D-ELSCM in terms of SE. Additionally, the corresponding average SE values are summarized in Table 4, which clearly shows that the proposed hyperchaotic map consistently achieves higher SE values than the other six chaotic systems. These results suggest that the 2D-ELSCM exhibits significantly greater complexity and randomness, essential characteristics for ensuring the security of cryptographic applications.

#### 3.2.6. 0–1 Test

The core idea of the 0–1 test is to analyze the relative positions of system trajectory points to determine whether the system exhibits chaotic behavior. If the trajectory points are randomly distributed in the phase space without any discernible regularity, the test value will approach 1, indicating chaotic dynamics. Conversely, if the trajectory demonstrates periodicity or stability, the test value will approach 0, indicating non-chaotic behavior.

The 0–1 test results, compared with those of other recent chaotic maps, are presented in Figure 6d, which underscores the robust performance of the proposed system. The average 0–1 test values are provided in Table 4, with the 2D-ELSCM achieving the highest mean value of 0.9970. This exceptional result highlights the superior chaotic dynamics of the 2D-ELSCM, further reinforcing its suitability for advanced cryptographic applications.

#### 3.2.7. NIST SP800-22 Test

The NIST SP800-22 test suite is a well-established statistical framework used to assess the randomness of pseudo-random number sequences, which is critical for cryptographic applications. The suite comprises 15 distinct sub-tests, each producing a *p*-value that reflects the statistical quality of the sequence. If the *p*-value of a sub-test exceeds 0.01, it indicates that the sequence has passed that particular test, confirming its randomness and unpredictability [30]. This ensures that the generated sequence is not easily predictable, a crucial requirement for secure encryption.

In this study, the NIST SP800-22 test results for the 2D-ELSCM are summarized in Table 5. As shown, the pseudo-random number sequences generated by the proposed 2D-ELSCM pass all 15 sub-tests successfully. The high *p*-values across all tests demonstrate that the generated sequences exhibit excellent randomness, which is essential for ensuring the unpredictability of the encryption process. Consequently, the 2D-ELSCM proves to be highly suitable for image cryptography, providing a robust and secure foundation for encrypting sensitive data.

## 4. Novel Chaotic Image Encryption Scheme

**Figure 7:**
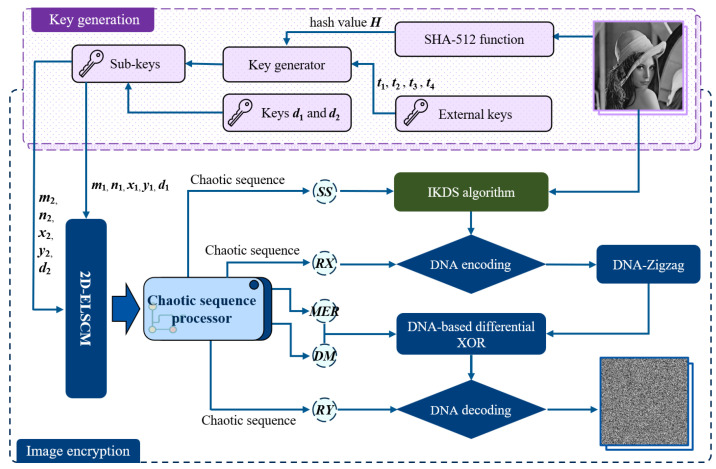
Framework of the proposed cryptographic scheme including key generation and encryption.

Based on the 2D-ELSCM, this study proposes an innovative chaotic image encryption method to ensure the security of image information. The framework of the proposed cryptographic scheme is depicted in Figure 7.

The algorithm consists of two main components: key generation and encryption. In the key generation phase, external keys {$t_{1},t_{2},t_{3},t_{4}$} within the interval (0,1) and the hash value of the plaintext image are jointly used to generate the sub-keys {$m_{1},n_{1},x_{1},y_{1},m_{2},n_{2},x_{2},y_{2}$} for iterating the 2D-ELSCM system. In addition, the parameters $d_{1}$ and $d_{2}$ are set to discard the initial chaotic sequence elements in order to eliminate transient effects and ensure the chaotic behavior of the generated sequences. This selection aims to achieve a substantial expansion of the key space and enhance the unpredictability of the generated sequences, while simultaneously maintaining a reasonable level of computational overhead. In the encryption phase, a scrambling and diffusion strategy is adopted to achieve high security. First, the IKDS algorithm is employed to scramble the pixel matrix of the plaintext image, where the shuffling indices are dynamically controlled by chaotic sequences to enhance the randomness of pixel positions. Subsequently, the scrambled image undergoes dynamic diffusion based on the DNA-Zigzag algorithm, in which the DNA rules are determined by chaotic sequences. This process not only increases the sensitivity to initial conditions but also enhances the algorithm’s resistance against common cryptographic attacks. The detailed encryption steps are described in the following subsections.

### 4.1. Key Generation Stage

To resist plaintext attacks, the proposed encryption scheme enhances the correlation with the original image by computing its hash value. External keys are then utilized to determine the initial values and parameters of the 2D-ELSCM. The specific steps are outlined below.

Step 1: The plaintext image serves as input to the SHA-512 hash function, generating a 512-bit hash value denoted as *H*.

Step 2: The hash value *H* is partitioned into 64 groups of decimal numbers, which are represented as $h_{1}$, $h_{2}$, $h_{3}$, …, $h_{64}$, respectively.

Step 3: The intermediate parameters $K_{1}$, $K_{2}$, $K_{3}$, $K_{4}$, $K_{5}$, and $K_{6}$ are generated by the following equations:

(2) $$\left\{\begin{array}{c} K_{1}=\frac{bxor\left(h_{1},h_{8}\right)+bxor\left(h_{57},h_{64}\right)+t_{3}}{2\times 256}\times t_{1}\\ K_{2}=\frac{bxor\left(h_{9},h_{16}\right)+bxor\left(h_{49},h_{56}\right)}{2\times 256}\times t_{2}\\ K_{3}=\frac{bxor\left(h_{17},h_{24}\right)+bxor\left(h_{25},h_{31}\right)}{2\times 256}\times t_{3}\\ K_{4}=\frac{bxor\left(h_{25},h_{32}\right)+bxor\left(h_{33},h_{44}\right)+bxor\left(h_{45},h_{56}\right)}{3\times 256}\times t_{1}\times t_{4}\\ K_{5}=\frac{max(h_{33},h_{40})}{sum(h_{33},h_{40})}\times t_{1}\times t_{2}\\ K_{6}=\frac{max(h_{41},h_{48})}{sum(h_{41},h_{48})}\times t_{3}\times t_{4} \end{array}\right.,$$

where $\left(t_{1},t_{2},t_{3},t_{4}\right)\in \left(0,1\right)$ are external keys. Additionally, bxor(*x*, *y*) denotes a function that calculates the XOR result between numbers *x* and *y*, max(*x*, *y*) represents the maximum value from *x* to *y*, and sum(*x*, *y*) calculates the total sum of values from *x* to *y*.

Step 4: Bring $K_{1}$, $K_{2}$, $K_{3}$, $K_{4}$, $K_{5}$, and $K_{6}$ into the following formula to obtain the initial values and the control parameters for 2D-ELSCM:

(3) $$\left\{\begin{array}{c} \begin{array}{cc} x_{1} & =mod\left(\left\lfloor \left(K_{2}+K_{3}+K_{4}\right)\times 10^{9}\right\rfloor \times t_{1}\times t_{4},\;512\right)/512\\ y_{1} & =mod\left(\left\lfloor \left(K_{1}+K_{4}+K_{5}\right)\times 10^{9}\right\rfloor \times t_{2}\times t_{3},\;512\right)/512\\ m_{1} & =mod\left(\left\lfloor \left(K_{1}+K_{2}+K_{3}+K_{6}\right)\times t_{1}\times t_{2}\times 10^{9}\right\rfloor ,\;10\right)+25\\ n_{1} & =mod\left(\left\lfloor \left(K_{1}+K_{3}+K_{5}+K_{6}\right)\times t_{3}\times t_{4}\times 10^{9}\right\rfloor ,\;10\right)+25 \end{array} \end{array}\right.,$$

(4) $$\left\{\begin{array}{c} \begin{array}{cc} x_{2} & =mod\left(\left\lfloor \left(K_{1}+K_{2}+K_{6}\right)\times 10^{9}\right\rfloor \times t_{1}\times t_{2},\;512\right)/512\\ y_{2} & =mod\left(\left\lfloor \left(K_{3}+K_{5}+K_{6}\right)\times 10^{9}\right\rfloor \times t_{2}\times t_{3},\;512\right)/512\\ m_{2} & =mod\left(\left\lfloor \left(K_{1}+K_{2}+K_{3}+K_{4}\right)\times t_{3}\times t_{4}\times 10^{9}\right\rfloor ,\;10\right)+25\\ n_{2} & =mod\left(\left\lfloor \left(K_{3}+K_{4}+K_{5}+K_{6}\right)\times t_{1}\times t_{4}\times 10^{9}\right\rfloor ,\;10\right)+25 \end{array} \end{array}\right.,$$

where $\left\lfloor \cdot \right\rfloor$ denotes the floor function, which returns the greatest integer less than or equal to the value inside, and $mod\left(\cdot \right)$ represents the modulo operation. Additionally, the parameters generated by Equation (3) are specifically employed in the scrambling step of the encryption process, enhancing the randomness of the image. Meanwhile, the parameters derived from Equation (4) are utilized in the diffusion step, ensuring a more uniform distribution of pixel values.

### 4.2. Encryption and Decryption Process

This study presents a robust encryption algorithm designed to ensure the security of image data. The following sections offer a comprehensive explanation of the scrambling and diffusion algorithms, along with a in-depth description of the specific encryption steps.

#### 4.2.1. Improved Knuth-Durstenfeld Shuffle Algorithm

Building upon the CKDS algorithm, the proposed improved Knuth-Durstenfeld shuffle (IKDS) algorithm integrates a chaotic system, leveraging the inherent unpredictability of chaotic sequences to control index selection during the shuffling process. This enhancement replaces conventional random number generation with indices derived from chaotic sequence values, thereby achieving permutations with improved randomness and security. Compared to traditional shuffling techniques, the incorporation of a chaotic system increases both the algorithm’s complexity and randomness, making it particularly suitable for security applications. The complete procedure of the IKDS algorithm is presented in Algorithm 2. Specifically, the algorithm traverses the array from the last element to the first. In each iteration, it computes an index by multiplying the corresponding chaotic sequence value by the current index plus one and applying the floor operation. Two elements at the current and computed positions are then swapped using a temporary variable. By utilizing the chaotic sequence in this manner, the IKDS algorithm ensures a high degree of unpredictability throughout the permutation process. Assuming the plaintext image $P_{1}$ has dimensions M×N, it is permuted following the steps outlined below.

Step 1: Input parameters $x_{1}$, $y_{1}$, $m_{1}$, and $n_{1}$, generated by Equation (3), into the 2D-ELSCM. Iterate the system $d_{1}+2\times M\times N$ times, discarding the first $d_{1}$ elements. Subsequently, chaotic sequences $X^{\prime }=\left\{X_{1}^{\prime },X_{2}^{\prime },\ldots ,X_{M\times N}^{\prime }\right\}$ and $Y^{\prime }=\left\{Y_{1}^{\prime },Y_{2}^{\prime },\ldots ,Y_{M\times N}^{\prime }\right\}$ are obtained.

Step 2: Modify the sequences $X^{\prime }$ and $Y^{\prime }$ to generate a combined sequence SS using Equation (5):

(5) $$SS\left(i\right)=\frac{X^{\prime }\left(i\right)+Y^{\prime }\left(i\right)}{2},$$

where *i* represents the *i*-th element of the sequence.

Step 3: Convert the 2D image matrix $P_{1}$ into a 1D sequence, which is then shuffled using Algorithm 2. Notably, this process relies on the chaotic sequence generated in Step 2 to ensure randomness. The resulting shuffled sequence is denoted as $P_{2}$.

Step 4: Transform the shuffled sequence back into a 2D format to restore the structural layout of the image. This output, denoted as $P_{3}$, is prepared for the subsequent diffusion step. At this stage, the scrambling process is complete.
**Algorithm 2** Pseudocode for IKDS algorithm**Require:** 
An array arr of length *n*; a chaotic sequence seq of length *n*; temporary variable temp;**Ensure:** 
A shuffled array arr1:**for** i=n−1 **to** 1 **do**2: j←⌊seq[i]×(i+1)⌋3: temp←arr[i]4: arr[i]←arr[j]5: arr[j]←temp6:**end for**7:**return** arr

#### 4.2.2. Dynamic DNA-Zigzag Diffusion Algorithm

Previous research integrated the Zigzag algorithm into the scrambling process, yielding satisfactory results. In this study, we further refine this approach by incorporating dynamic DNA encoding and extending its application to the diffusion process.

The proposed DNA-Zigzag method merges DNA encoding with Zigzag scanning techniques to enhance the security and randomness of image encryption. Initially, the image data is transformed into a DNA sequence and organized into a matrix. Subsequently, a new sequence is generated through Zigzag scanning. This approach significantly increases encryption complexity, making it well-suited for applications in secure communication and data storage, thereby addressing the growing demand for high-security encryption. The detailed encryption steps are outlined below, with the core algorithm presented in Algorithm 3.
**Algorithm 3** Implementation process of the DNA-Zigzag**Require:** 
A DNA sequence *seq*, dimensions *height* and *width*1:n←lengthofseq2:**if** width×height≠n **then**3:  **Error**4:**end if**5:*matrix* ← 2D array of size height×width6:*zigzag_seq* ← empty list7:**for** i=0 to height−1 **do**8: **for** j=0 to width−1 **do**9:  matrix[i][j]←seq[i×width+j]10: **end for**11:**end for**12:**for** i=0 to height−1 **do**13: **if** imod2=0 **then**14:  **for** j=0 to width−1 **do**15:      *zigzag_seq.append*(matrix[i][j])16:  **end for**17: **else**18:  **for** j=width−1 to 0 by −1 **do**19:      *zigzag_seq.append*(matrix[i][j])20:  **end for**21: **end if**22:**end for**23:**return** *zigzag_seq*

Step 1: Input parameters $x_{2}$, $y_{2}$, $m_{2}$, and $n_{2}$, generated by Equation (4), into the 2D-ELSCM. Iterate the system $d_{2}+4\times M\times N$ times. Discard the first $d_{2}$ elements from the resulting sequences, and obtain the chaotic sequences XX, YY, WW, and ZZ, each with a length of M×N.

Step 2: To further enhance the complexity and security of the encryption algorithm, multiple chaotic sequences are generated using Equation (6):

(6) $$\left\{\begin{array}{c} RX\left(i\right)=mod\left(\left\lfloor xx\left(i\right)\times 10^{15}\right\rfloor ,8\right)\\ RY\left(i\right)=mod\left(\left\lfloor zz\left(i\right)\times 10^{15}\right\rfloor ,8\right)\\ MER\left(i\right)=mod\left(\left\lfloor ww\left(i\right)\times 10^{15}\right\rfloor ,8\right)\\ DM\left(i\right)=mod\left(\left\lfloor yy\left(i\right)\times 10^{15}\right\rfloor ,256\right) \end{array}\right.,$$

where xx, yy, ww, and zz represent elements from the chaotic sequences XX, YY, WW, and ZZ, respectively, and *i* denotes the *i*-th element of these sequences.

Step 3: Convert the input image matrix $P_{4}$ into a binary sequence. Then, utilize the elements from the chaotic sequence RX as encoding rules to dynamically encode the binary sequence into a DNA sequence $D_{1}$.

Step 4: In this step, the dynamic DNA-Zigzag diffusion process, as detailed in Algorithm 3, is applied to the DNA sequence, where the sequence undergoes a transformation that rearranges its elements based on the zigzag pattern, ultimately generating a disordered version of the original sequence, which is denoted as $D_{2}$.

Step 5: DNA mask and differential XOR operation. The sequence MER is used as the mask encoding rule to encode the DM into sequence mk. Subsequently, the $D_{3}$ is obtained by XORing mk with $D_{2}$ specified in Table 2 and calculated using Equation (7). This ensures the diffusion process is interdependent among the chaotic sequences:

(7) $$\left\{\begin{array}{cc} D_{3}\left(i\right)=D_{2}\left(i\right)\;⊕\;mk\left(i\right)\;, & i=1\\ D_{3}\left(i\right)=D_{2}\left(i\right)\;⊕\;mk\left(i\right)\;⊕\;D_{3}\left(i-1\right)\;, & i\neq 1 \end{array}\right.,$$

where $D_{3}\left(i\right)$, $D_{2}\left(i\right)$, and mk(i) denote the *i*-th elements of the sequences $D_{3}$, $D_{2}$, and mk, respectively.

Step 6: Decode the DNA ciphertext $D_{4}$ into a binary sequence using the chaotic sequence RY. Subsequently, convert the binary sequence into a grayscale image to reconstruct the original image structure, yielding the ciphertext image $C_{1}$.

Up to this point, the encryption algorithm has been fully described. The decryption process, serving as the inverse of the encryption, recovers the original image using the correct key. Therefore, a detailed discussion of the decryption procedure is omitted for brevity. It is important to note that for color images, the R, G, and B channels are separated, independently encrypted, and then recombined to produce the final encrypted color image.

## 5. Experimental Simulation and Security Analysis

### 5.1. Simulation Environment and Results

The proposed encryption scheme was implemented using Python 3.8.1 within the PyCharm 2023.1.3 (Community Edition) development environment. The implementation was carried out on a system equipped with 16.00 GB of RAM and an Intel(R) Core(TM) i5-12600 CPU running at 3.30 GHz, operating on the Windows 10 platform. This section presents a performance evaluation of the proposed encryption algorithm from several perspectives. Additionally, a summary of the external keys and other experimental parameters utilized in the simulations is provided in Table 6.

### 5.2. Histogram Analysis

In the domain of image encryption, histogram analysis serves as a critical method for assessing the effectiveness of encryption algorithms. An ideal encryption algorithm should significantly alter the pixel distribution of the original image, producing a ciphertext image with a uniformly distributed histogram to ensure privacy and unpredictability.

**Figure 8:**
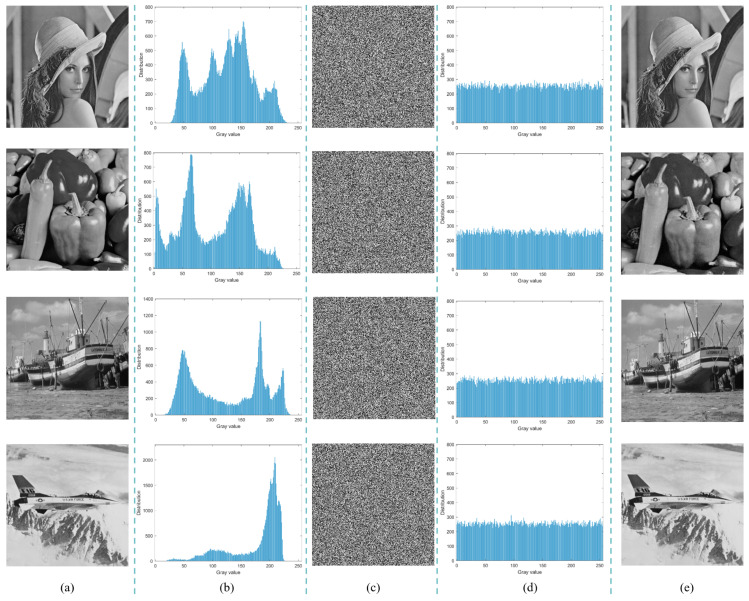
Simulation test of encryption algorithm: (**a**) plaintext images (256 × 256); (**b**) histograms of the plain images; (**c**) cipher images; (**d**) histograms of the ciphertext images; (**e**) decrypted images.

Figure 8 displays the original image, the encrypted image, and their corresponding histograms. As shown in Figure 8a,c,e, the proposed algorithm effectively transforms the original image into a noise-like structure while enabling its lossless reconstruction. Additionally, in Figure 8b,d, the histogram of the ciphertext image exhibits a uniform distribution, which contrasts sharply with the plaintext histogram. This result highlights the algorithm’s ability to disrupt the statistical properties of the image, thereby rendering the ciphertext image highly resistant to analysis through basic statistical methods.

### 5.3. Chi-Square Test

In the field of image encryption, the Chi-square ($\chi^{2}$) test is commonly utilized to evaluate the statistical characteristics of encrypted images. This test assesses whether the encryption algorithm effectively disrupts the original pixel distribution, ensuring randomness and uniformity in the ciphertext image. The Chi-square statistic $\chi^{2}$ is mathematically expressed as [12]:

(8) $$\chi^{2}=\sum_{i=1}^{256}\frac{{\left(f_{u}-f_{v}\right)}^{2}}{f_{v}},$$

where *i* denotes the number of gray levels, while $f_{u}$ and $f_{v}$ represent the observed and expected frequencies, respectively. The histogram distribution is deemed uniform if the Chi-square value falls below 293.24783. Table 7 summarizes the Chi-square values for 256 × 256 plaintext and ciphertext images. Notably, the Chi-square values of the ciphertext images are significantly lower than those of the plaintext images, and all ciphertext values satisfy the criteria for passing the Chi-square test.

### 5.4. Correlation Analysis

Correlation analysis is a crucial technique for assessing the security of image encryption algorithms. It quantifies the extent to which the encryption process disrupts the statistical properties of an image by calculating the correlation coefficients between adjacent pixels in the horizontal, vertical, and diagonal directions. The correlation coefficient $CC_{xy}$ between adjacent pixels *x* and *y* is defined as follows [13]:

(9) $$CC_{xy}=\frac{\sum_{i=1}^{N}\left(x_{i}-\bar{x}\right)\left(y_{i}-\bar{y}\right)}{\sqrt{\sum_{i=1}^{N}{\left(x_{i}-\bar{x}\right)}^{2}\sum_{i=1}^{N}{\left(y_{i}-\bar{y}\right)}^{2}}},$$

where *N* denotes the total number of pixels, and $\bar{x}$ and $\bar{y}$ represent the mean values of *x* and *y*, respectively. From Equation (9), it is evident that the correlation coefficients $CC_{xy}$ lie within the range [0, 1]. Original images typically exhibit high correlation, whereas an ideal encrypted image should display correlation coefficients close to 0, indicating that the correlations between adjacent pixels have been effectively eliminated.

**Figure 9:**
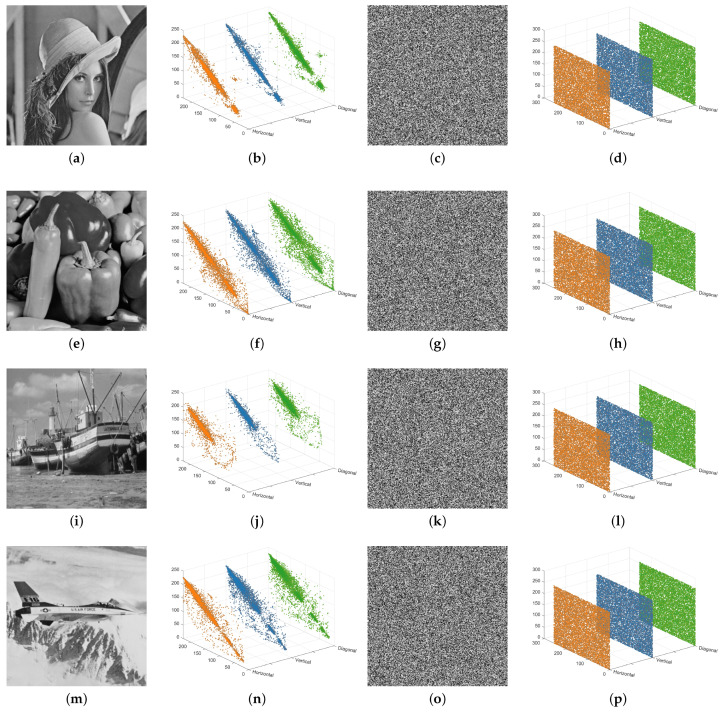
Correlation distributions: (**a**,**e**,**i**,**m**) plain images; (**b**,**f**,**j**,**n**) histograms of those plain images in three directions; (**c**,**g**,**k**,**o**) cipher images of (**a**,**e**,**i**,**m**); (**d**,**h**,**l**,**p**) histograms of those cipher images in three directions.

The 3D diagrams of correlation coefficients in three directions for both the test images and their encrypted counterparts are shown in Figure 9. As illustrated, the correlation in all directions is uniformly distributed across the entire coordinate space. Furthermore, the correlation analysis results of various methods are presented in Table 8. It is evident that the correlation coefficients of the ciphertext images generated by the proposed algorithm are closer to 0 compared to those of other algorithms, demonstrating its enhanced resistance to statistical attacks.

### 5.5. Differential Attack Analysis

The Number of Pixels Change Rate (NPCR) and Unified Average Changing Intensity (UACI) are widely used metrics for evaluating the ability of encryption algorithms to resist differential attacks. The mathematical expressions for NPCR and UACI are given as follows [34]:

(10) $$\mathrm{NPCR}=\frac{1}{M\times N}\sum_{i=1}^{M}\sum_{j=1}^{N}\left|\eta \left(I_{1}\left(i,j\right)-\left(I_{2}\left(i,j\right)\right)\right)\right|\times 100\%,$$

(11) $$\mathrm{UACI}=\frac{1}{M\times N}\sum_{i=1}^{M}\sum_{j=1}^{N}\frac{I_{1}\left(i,j\right)-I_{2}\left(i,j\right)}{F}\times 100\%,$$

(12) $$\begin{array}{c} \eta \left(x\right)=\left\{\begin{array}{cc} 1, & x>0\\ 0, & x=0\\ -1, & x<0 \end{array}\right., \end{array}$$

where $I_{1}$ and $I_{2}$ denote the cipher images of the original plaintext image and the plaintext image with one pixel altered, respectively. *M* and *N* are the height and width of the test images, and *F* is the maximum allowable pixel value of the image. Additionally, the ideal values for the NPCR and UACI tests are 99.6094% and 33.4635%, respectively.

A novel evaluation criterion for the NPCR and UACI tests was introduced by [35]. The NPCR test is considered successful if the NPCR value exceeds the one-sided hypothesis threshold determined by the significance level (α). This can be expressed as follows:

(13) $$NPCR_{\alpha }^{*}=\frac{F-\Phi^{-1}\left(\alpha \right)\sqrt{\frac{F}{M\times N}}}{\left(F+1\right)},$$

where $\Phi^{-1}\left(\alpha \right)$ represents the inverse cumulative density function (CDF) of the standard normal distribution. Meanwhile, α denotes the test level and it is set to 0.01 and 0.05 in subsequent testing scenarios [4].

In a similar manner, the UACI test is deemed successful if the calculated UACI value lies within the range of (${\mathrm{UACI}}_{\alpha }^{*-}$, ${\mathrm{UACI}}_{\alpha }^{*+}$), where ${\mathrm{UACI}}_{\alpha }^{*-}$ and ${\mathrm{UACI}}_{\alpha }^{*+}$ are specified as

(14) $$\left\{\begin{array}{c} {\mathrm{UACI}}_{\alpha }^{*-}=\mu_{u}-\Phi^{-1}\left(\alpha /2\right)\sigma_{u}\\ {\mathrm{UACI}}_{\alpha }^{*+}=\mu_{u}+\Phi^{-1}\left(\alpha /2\right)\sigma_{u}\\ \mu_{u}=\left(F+2\right)/\left(3F+3\right)\\ \sigma_{u}^{2}=\left(F+2\right)\left(F^{2}+2F+3\right)/18{\left(F+1\right)}^{2}MNF \end{array}\right..$$

In this subsection, several test images with resolutions of 256×256 and 512×512 were selected to evaluate the algorithm’s sensitivity to minor modifications. For each image, 100 trials were conducted by randomly selecting a pixel and increasing its value by 1. The NPCR and UACI results under different values of α are summarized in Table 9 and Table 10. When α=0.05, the pass rates for both metrics exceed 90%; when α=0.01, the pass rates approach 100%. The proposed algorithm achieves average NPCR and UACI values that are very close to the ideal theoretical values. Furthermore, Table 11 presents a detailed comparison of NPCR and UACI values across different test images. The proposed method consistently outperforms or matches the performance of the approaches reported in [4,36], further confirming its robustness against differential attacks.

### 5.6. Information Entropy

Information entropy reflects the randomness in the distribution of pixel intensity values in an image. For an ideally encrypted image, the entropy approaching its theoretical maximum value. The formula for information entropy *H* is given by:

(15) $$H\left(m\right)=-\sum_{i=1}^{N}p(m_{i})\log P\left(m_{i}\right),$$

where $P\left(m_{i}\right)$ represents the probability of pixel intensity $m_{i}$, and *N* denotes the number of intensity levels, which is commonly set to 256. When the information entropy of an encrypted image approaches the theoretical maximum of 8, it indicates a nearly uniform and random distribution of pixel intensities [32]. To comprehensively evaluate the encryption performance, images of different sizes were selected to calculate the information entropy values for both plaintext and ciphertext images, as defined in Equation (15). Table 12 presents the entropy results for grayscale and color images with resolutions of 256 × 256 and 512 × 512, respectively. The ciphertext images exhibit entropy values consistently close to the theoretical maximum value of 8, indicating that the proposed encryption algorithm effectively disrupts the original pixel distribution and demonstrates good applicability and encryption performance for both grayscale and color images. To provide a more comprehensive evaluation of the encryption performance, a comparative analysis of information entropy across two types of images was conducted. As summarized in Table 13 and Table 14, the proposed scheme consistently achieves comparable or higher entropy values for ciphertext images relative to most state-of-the-art methods, thereby demonstrating improved randomness and robustness. These results highlight the scheme’s effectiveness in enhancing the security of encrypted image data against potential cryptographic attacks.

### 5.7. Key Analysis

Key analysis is a vital component in evaluating the performance of an encryption algorithm. This section will validate the security and robustness of the algorithm from two key aspects: key space and key sensitivity.

#### 5.7.1. Key Space Analysis

The key space is a crucial metric for assessing the security of an encryption algorithm, as its size directly impacts the algorithm’s resistance to brute-force attacks. An encryption algorithm with a sufficiently large key space can effectively thwart attackers attempting to decrypt data by exhaustively searching all possible keys. According to [38], the key space should exceed $2^{100}$ to guarantee the security of encryption algorithm.

The proposed encryption algorithm features a robust key design, consisting of the following components: (i) 512-bit hash key generated by the original image; (ii) the external parameter $t_{1}$, $t_{2}$, $t_{3}$, $t_{4}$; and (iii) the abandoning number $d_{1}$ and $d_{2}$ of chaotic sequences. Assuming that the calculation accuracy in this experimental environment is $10^{-15}$, the key space of this algorithm is $2^{512}\times 10^{60}\times 10^{8}\approx 2^{732}$. The key space of the proposed algorithm is compared with that of other schemes, and the results are presented in Table 15. The results clearly demonstrate that the proposed algorithm achieves the largest key space, significantly enhancing security and resistance to brute-force attacks.

#### 5.7.2. Key Sensitivity Analysis

An encryption algorithm with high sensitivity ensures that even a slight change in the key results in completely different encrypted images, making it difficult for attackers to recover the original image or obtain any information about the key. To evaluate key sensitivity quantitatively, the number of bit change rate (NBCR) [40] is utilized. For two bit sequences $\xi_{1}$ and $\xi_{2}$ of length $L_{\xi }$, the NBCR is determined using:

(16) $$NBCR\left(\xi_{1},\xi_{2}\right)=\frac{Hm\left[\xi_{1},\xi_{2}\right]}{L_{\xi }}\times 100\%,$$

where *Hm*$\left[\xi_{1},\xi_{2}\right]$ is the Hamming distance of $\xi_{1}$ and $\xi_{2}$. If the sequences $\xi_{1}$ and $\xi_{2}$ are approximately independent, the NBCR value will approach 50%. The test process for key sensitivity is as follows:

(i) The original SHA-512 key, denoted as *K*, is initially prepared, and a modified version $K_{m}$ is derived by altering a single bit in *K* to evaluate sensitivity.

(ii) The encryption of the original image is performed with both *K* and $K_{m}$, generating ciphertext images $C_{1}\left(i\right)$ and $C_{2}\left(i\right)$ (i=1,2,3,…,512). The normalized bit change rate (NBCR) $\mathrm{NBCR}(C_{1}\left(i\right),C_{2}\left(i\right))$ between the ciphertexts is calculated to measure key sensitivity.

(iii) The decryption of both ciphertext sets is carried out using $K_{m}$, resulting in decrypted images $D_{1}\left(i\right)$ and $D_{2}\left(i\right)$ (i=1,2,3,…,512). The $\mathrm{NBCR}(D_{1}\left(i\right),D_{2}\left(i\right))$ values are then evaluated to verify the algorithm’s sensitivity during decryption.

**Figure 10:**
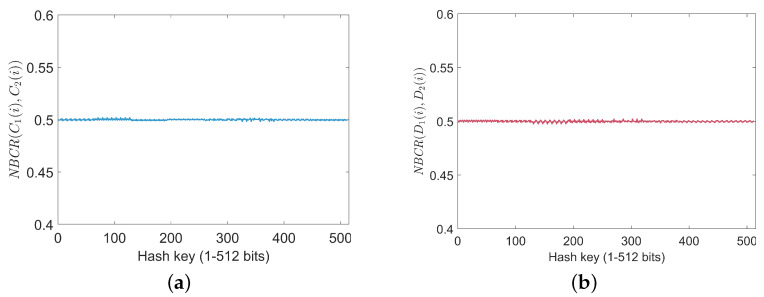
NBCR analysis for encrypted and decrypted images: (**a**) NBCR of encrypted images with a one-bit key change; (**b**) NBCR of decrypted images with a one-bit key change.

As illustrated in Figure 10, the NBCR value curves for both ciphertext and decrypted images remain approximately 50%. This observation highlights the proposed encryption scheme’s high key sensitivity and its ability to prevent unauthorized access through minor key modifications.

### 5.8. Robustness Analysis

Image transmission and storage processes are often subject to various forms of interference and attacks. A robust image encryption algorithm is expected to effectively resist these disturbances and environmental variations, ensuring that the decrypted image closely resembles the original. Evaluations of the proposed algorithm’s robustness are conducted through noise and cropping attack analyses.

#### 5.8.1. Noise Attack Analysis

**Figure 11:**
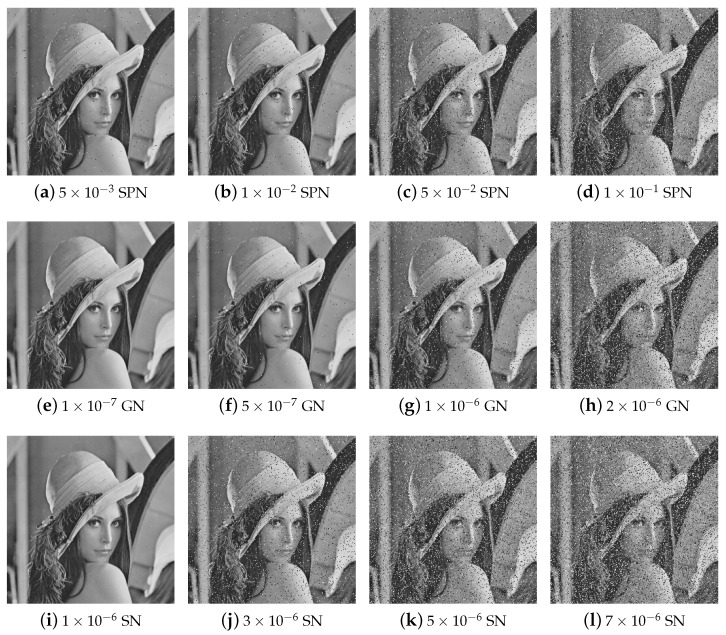
Decryption results of cipher images with different densities of SPN, GN, and SN.

Resistance to noise attacks is a critical attribute of a robust image encryption algorithm, enabling successful decryption despite interference. In this study, the application of salt-and-pepper noise (SPN), Gaussian noise (GN), and speckle noise (SN) to encrypted images simulates realistic transmission scenarios involving noise attacks. The decrypted results from noisy images are displayed in Figure 11. Observations reveal that, even with added noise, the decryption process effectively restores the original images with high visual quality.

#### 5.8.2. Data Loss Attack Analysis

During image transmission, interference within the channel often causes significant data loss, posing challenges to data integrity. The evaluation of an encryption algorithm’s robustness against cropping attacks is therefore essential to assess its performance under such conditions.

**Figure 12:**
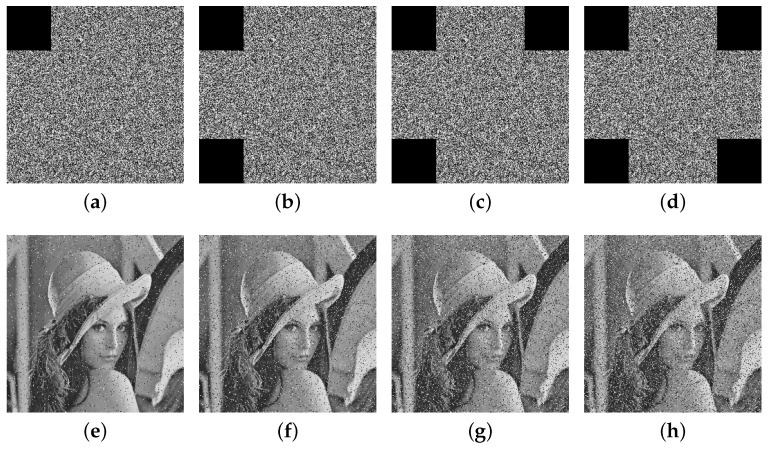
The encryption images with cropping attack (**a**) 1/16, (**b**) 1/8, (**c**) 3/16, (**d**) 1/4 and (**e**–**h**) represent their respective decrypted images.

**Figure 13:**
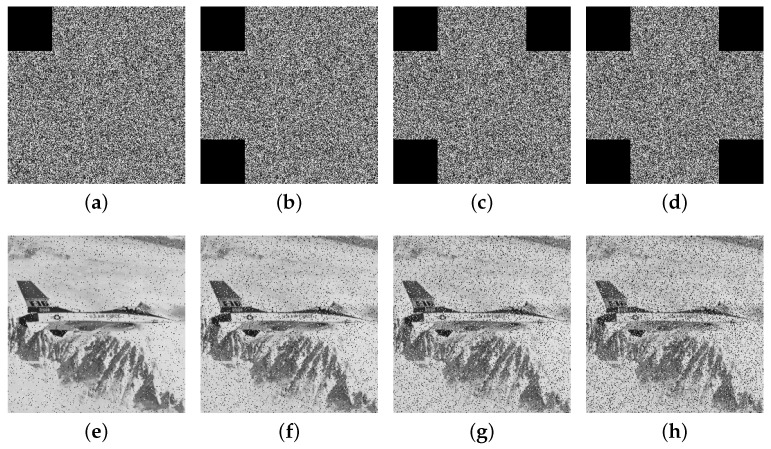
The encryption images with cropping attack (**a**) 1/16, (**b**) 1/8, (**c**) 3/16, (**d**) 1/4 and (**e**–**h**) represent their respective decrypted images.

Figure 12 and Figure 13 present the restoration results of two test cipher images subjected to pixel block losses of 1/16, 1/8, 3/16, and 1/4, respectively. The results indicate that, despite varying degrees of data loss, a substantial portion of the original image’s information remains visible in the decrypted outputs. These findings highlight the proposed algorithm’s strong capability to preserve critical information and resist cropping attacks effectively.

### 5.9. Time Complexity Analysis

To validate its practical value, an encryption algorithm should minimize time complexity without compromising security. This subsection evaluates the time complexity of the proposed approach.

For a plain image of size m×n, the computational complexity of the proposed algorithm primarily arises from the operations in the permutation and diffusion stages. During the shuffle permutation process, the first step involves generating two chaotic sequences, resulting in a computational complexity of O(2×m×n). The second step modifies these chaotic sequences, contributing a time complexity of O(2×m×n). The third step performs the IKDS algorithm, which has a time complexity of O(m×n).

The time complexity analysis for the DNA-based diffusion process is as follows. In the first step, chaotic sequences are generated by iterating the 2D-ELSCM model to produce four sequences, leading to a time complexity of O(4×m×n). The subsequent step modifies these chaotic sequences, also contributing a time complexity of O(4×m×n). Steps 3 and 6 involve DNA encoding and decoding operations, each with a time complexity of O(m×n). Step 4 executes the DNA-zigzag operation, with a time complexity of O(4×m×n). Finally, Step 5 performs the DNA differential XOR operation, which has a time complexity of O(m×n).

As a result, the overall time complexity of the proposed encryption scheme is O(20×m×n). To further verify the practical efficiency of the proposed algorithm, the actual encryption and decryption times were measured and compared with existing methods. As shown in Table 16, the proposed algorithm achieves encryption and decryption times that are comparable to those of reference [4] and significantly lower than those reported in reference [34]. These results demonstrate that the proposed approach not only ensures robust security but also maintains high computational efficiency, making it suitable for real-time and resource-constrained applications.

## 6. Conclusions

This study presents a novel 2D hyperchaotic map, referred to as the 2D-ELSCM, developed by integrating exponential, logarithmic, and sine functions. Its chaotic characteristics were thoroughly evaluated, yielding an LLE of 8.3175, PE of 0.9998, SE of 1.9826, KE of 2.1117, and a 0–1 test result of 0.9970. Furthermore, the 2D-ELSCM successfully passed the NIST randomness tests, collectively confirming its strong randomness and unpredictability.

Building upon the 2D-ELSCM, an advanced image encryption scheme was developed. It integrates an IKDS algorithm to disrupt local pixel correlations, and a dynamic diffusion mechanism combining DNA encoding and the Zigzag transform to achieve global pixel diffusion. Key sensitivity and space are enhanced by deriving initial conditions from the SHA-512 hash of the plaintext image combined with an external key. Notably, the scheme is applicable to both grayscale and color images, with color encryption performed through independent processing of the R, G, and B channels. The encrypted color images also exhibit excellent randomness, ensuring the robustness and effectiveness of the proposed method in practical scenarios. Extensive simulations validate the scheme’s effectiveness, achieving correlation coefficients close to 0, an average NPCR of 99.6090%, and UACI of 33.4707%, thereby demonstrating strong resistance against differential attacks and excellent encryption quality for secure image transmission and storage.

Although the proposed method exhibits robust security and efficiency, further improvements are planned. Future work will focus on enhancing diffusion unpredictability by employing chaotic sequences to generate nonlinear, fluctuating Zigzag paths, thereby surmounting the constraints of fixed patterns. Moreover, the integration of digital signature techniques and key exchange protocols will be explored to further strengthen the security of key generation and transmission, ultimately contributing to a more secure and adaptable encryption framework. 

## Figures and Tables

**Table 1:** Rules of DNA encoding and decoding.

Rule	1	2	3	4	5	6	7	8
**00**	A	A	T	T	G	G	C	C
**01**	C	G	C	G	T	A	T	A
**10**	G	C	G	C	A	T	A	T
**11**	T	T	A	A	C	C	G	G

**Table 2:** XOR operations of DNA coding.

⊕	A	G	C	T
**A**	A	T	C	G
**G**	G	A	T	C
**C**	C	G	A	T
**T**	T	C	G	A

**Table 3:** Comparison among the existing 2D chaotic systems.

Maps	Mathematical Definition	Parameters
2D-SICM [17]	$\left\{\begin{array}{c} x_{i+1}=\alpha sin\left(\pi \left(siny_{i}+cosx_{i}\right)\right)\\ y_{i+1}=sin\left(\frac{\beta }{sinx_{i}+cosy_{i}}\right) \end{array}\right.$	α, β
2D-ICM [23]	$\left\{\begin{array}{c} x_{i+1}=sin\left(\frac{a}{y_{i}}\right)sin\left(\frac{b}{x_{i}}\right)\\ y_{i+1}=sin\left(\frac{a}{x_{i}}\right)sin\left(\frac{b}{y_{i}}\right) \end{array}\right.$	*a*, *b*
2D-CLSS [24]	$\left\{\begin{array}{c} x_{i+1}=sin\left(\pi py_{i}\left(1-y_{i}\right)\right)\\ y_{i+1}=sin\left(\pi \left(x_{i}+y_{i}\right)\right) \end{array}\right.$	*p*
2D-ICSCM [25]	$\left\{\begin{array}{c} x_{i+1}=sin\left(\frac{\alpha }{siny_{i}}\right)\\ y_{i+1}=\beta sin\left(\pi \left(x_{i}+y_{i}\right)\right) \end{array}\right.$	α, β
2D-HCM [26]	$\left\{\begin{array}{c} x_{i+1}=sin\left(\frac{h\pi }{siny_{i}}\right)\\ y_{i+1}=rsin\left(\pi x_{i}y_{i}\right) \end{array}\right.$	*h*, *r*
2D-LSM [27]	$\left\{\begin{array}{c} x_{i+1}=cos\left(4ax_{i}\left(1-x_{i}\right)+bsin\left(\pi y_{i}\right)+1\right)\\ y_{i+1}=cos\left(4ay_{i}\left(1-y_{i}\right)+bsin\left(\pi x_{i}\right)+1\right) \end{array}\right.$	*a*, *b*

**Table 4:** Comparative analysis of the average chaotic metrics for various 2D chaotic maps.

Items	2D-SICM [17]	2D-ICM [23]	2D-CLSS [24]	2D-ICSCM [25]	2D-HCM [26]	2D-LSM [27]	2D-ELSCM
LLE	5.3788	6.3030	2.7787	5.1301	6.8962	5.4899	8.3175
PE	0.9678	0.9236	0.9989	0.9794	0.9970	0.9947	0.9998
SE	1.8816	1.7540	1.7409	1.7718	0.9282	1.8938	1.9826
KE	1.6817	1.6743	1.9734	1.9323	1.4427	1.8341	2.1117
0–1 test	0.8959	0.9797	0.9560	0.7389	0.9867	0.9618	0.9970

**Table 5:** NIST randomness test results of 2D-ELSCM.

No.	Test Items	*p*-Value	Results
1	ApproximateEntropy	0.401199	Pass
2	BlockFrequency	0.437274	Pass
3	CumulativeSums	0.647169	Pass
4	FFT	0.719747	Pass
5	Frequency	0.883171	Pass
6	LinearComplexity	0.657933	Pass
7	LongestRunOfOnes	0.334538	Pass
8	NonOverlappingTemplate	0.503998	Pass
9	OverlappingTemplate	0.032923	Pass
10	RandomExcursions	0.370023	Pass
11	RandomExcursionsVariant	0.402356	Pass
12	Rank	0.595549	Pass
13	Runs	0.851383	Pass
14	Serial	0.253076	Pass
15	Universal	0.224821	Pass

**Table 6:** Experimental parameters and values.

Parameters	Values
The external parameters	$t_{1}=0.1673827831$
	$t_{2}=0.5410551238$
	$t_{3}=0.3123129594$
	$t_{4}=0.4506314590$
Discarding number of sequence	$d_{1}=3000$, $d_{2}=5000$

**Table 7:** Chi-square $\chi^{2}$ values for test plaintext and ciphertext images.

Images	Plaintext Images	Ciphertext Images	Result
Lena	3.9713 × $10^{4}$	263.0622	pass
Peppers	3.5765 × $10^{4}$	244.5722	pass
Airplane	1.76038 × $10^{5}$	206.0125	pass
Lake	4.9386 × $10^{4}$	235.2182	pass
Boat	1.00282 × $10^{5}$	236.0743	pass
5.1.10	5.0664 × $10^{4}$	256.8210	pass
5.1.11	2.19986 × $10^{5}$	258.8132	pass
5.1.12	2.80960 × $10^{5}$	231.1872	pass
5.1.13	1.1936401 × $10^{7}$	262.0661	pass
5.1.14	5.0130 × $10^{4}$	227.4830	pass
5.1.15	5.6809 × $10^{4}$	266.7975	pass

**Table 8:** Correlation coefficients of different images using various algorithms.

Images	Algorithms	Horizontal	Vertical	Diagonal
Lena	Plaintext image	0.9214	0.9595	0.8978
	Ours	−0.0007	0.0005	−0.001
	Ref. [4]	0.0053	0.0059	0.0031
	Ref. [31]	−0.0021	−0.0012	0.0017
	Ref. [32]	0.006	0.0021	−0.0005
	Ref. [33]	−0.0006	0.0024	−0.0002
Peppers	Plaintext image	0.9633	0.9743	0.9421
	Ours	−0.0008	−0.0002	−0.0001
	Ref. [31]	0.0005	0.0004	0.0032
	Ref. [32]	−0.0025	−0.004	−0.0015
	Ref. [33]	−0.0009	0.0044	0.0002
Boat	Plaintext image	0.9255	0.9411	0.8795
	Ours	0.0006	0.0025	0.0002
	Ref. [31]	0.0023	0.0032	0.0016
	Ref. [32]	−0.0068	0.0041	−0.0044
Airplane	Plaintext image	0.9390	0.9360	0.8946
	Ours	−0.0014	0.0024	−0.0006
	Ref. [31]	0.0038	−0.0048	−0.0001
	Ref. [32]	0.0023	−0.0013	0.0037
Baboon	Plaintext image	0.8401	0.7789	0.7448
	Ours	0.0003	0.0011	−0.0005
	Ref. [31]	0.0015	0.0048	0.0016
	Ref. [32]	0.0005	0.0051	−0.0034
	Ref. [33]	−0.001	0.0019	0.0001

**Table 9:** NPCRs of different images using the proposed scheme.

Images	Maximum (%)	Minimum (%)	Mean (%)	Pass Rate (%)	
				${\mathrm{NPCR}}_{0.05}^{*}=99.5693\%$	${\mathrm{NPCR}}_{0.01}^{*}=99.5527\%$
Lena (256 × 256)	99.6811	99.5407	99.6097	94	99
Peppers	99.6811	99.5514	99.6103	96	99
Barbara	99.6704	99.5529	99.6101	94	100
Lake	99.6674	99.5544	99.6094	96	100
Airplane	99.6887	99.5483	99.6083	93	98
Couple	99.6567	99.5453	99.6098	95	99
5.1.09	99.6628	99.5438	99.6120	97	98
5.1.10	99.6719	99.5361	99.6066	92	99
5.1.11	99.6811	99.5621	99.6147	98	100
5.1.12	99.6750	99.5514	99.6125	93	99
5.1.13	99.6628	99.5483	99.6071	92	99
5.1.14	99.6735	99.5331	99.6100	94	97
7.1.01 (512 × 512)	99.6349	99.5446	99.6101	93	99
7.1.02	99.6422	99.5427	99.6102	97	98
7.1.03	99.6403	99.5534	99.6103	98	100
7.1.04	99.6437	99.5442	99.6096	96	98
7.1.05	99.6437	99.5542	99.6093	95	100
7.1.06	99.6433	99.5501	99.6092	97	99
7.1.07	99.6449	99.5511	99.6081	95	98
7.1.08	99.6323	99.5469	99.6093	94	97
7.1.09	99.6311	99.5531	99.6093	95	99
7.1.10	99.6487	99.5580	99.6090	94	100
Average	99.6423	99.5364	99.6090	96	99

**Table 10:** UACIs of different images using the proposed scheme.

Images	Maximum (%)	Minimum (%)	Mean (%)	Pass Rate (%)	
				${\mathrm{UACI}}_{0.05}^{*-}=33.2834\%$	${\mathrm{UACI}}_{0.01}^{*-}=33.2255\%$
				${\mathrm{UACI}}_{0.05}^{*+}=33.6447\%$	${\mathrm{UACI}}_{0.01}^{*+}=33.7016\%$
Lena (256 × 256)	33.6203	33.1533	33.4680	96	99
Peppers	33.6013	33.2386	33.4248	92	100
Barbara	33.7119	33.1774	33.4511	93	97
Lake	33.6580	33.3253	33.5176	96	100
Airplane	33.6711	33.2191	33.4469	94	99
Couple	33.7660	33.3094	33.5120	95	98
5.1.09	33.6157	33.3026	33.4671	100	100
5.1.10	33.6442	33.2350	33.4363	96	100
5.1.11	33.6555	33.1720	33.4228	93	99
5.1.12	33.6846	33.2265	33.4686	92	100
5.1.13	33.6376	33.2388	33.4649	98	100
5.1.14	33.6822	33.2957	33.4902	98	100
7.1.01 (512 × 512)	33.5230	33.3321	33.4509	96	98
7.1.02	33.5641	33.3022	33.4647	99	99
7.1.03	33.5544	33.3573	33.4538	94	100
7.1.04	33.5706	33.3450	33.4614	97	99
7.1.05	33.5649	33.3573	33.4665	94	97
7.1.06	33.5979	33.3811	33.4867	91	98
7.1.07	33.5454	33.3352	33.4889	93	98
7.1.08	33.6071	33.3045	33.4484	95	97
7.1.09	33.5279	33.3296	33.4549	91	98
7.1.10	33.5532	33.3483	33.4284	97	100
Average	33.6218	33.2774	33.4707	95	98

**Table 11:** Comparison of the NPCR and UACI values using various algorithms.

Images	NPCR (%)			UACI (%)		
	Ref. [4]	Ref. [36]	Ours	Ref. [4]	Ref. [36]	Ours
5.1.09 (256 × 256)	99.6094	99.5811	99.6120	33.4817	33.4614	33.4671
5.1.10	99.6185	99.6048	99.6066	33.4672	33.4758	33.4363
5.1.11	99.6048	99.6063	99.6147	33.4216	33.5633	33.4228
5.1.12	99.6094	99.6323	99.6125	33.4362	33.5595	33.4686
5.1.13	99.6109	99.6109	99.6071	33.4393	33.4411	33.4649
5.1.14	99.6002	99.5865	99.6100	33.4646	33.5315	33.4902
7.1.01 (512 × 512)	99.6071	99.6086	99.6101	33.4632	33.4915	33.4509
7.1.02	99.6094	99.6071	99.6102	33.4572	33.5495	33.4647
7.1.03	99.6098	99.6273	99.6103	33.4241	33.4768	33.4538
7.1.04	99.6117	99.6269	99.6096	33.4671	33.4754	33.4614
7.1.05	99.6105	99.6082	99.6093	33.4469	33.4721	33.4665
7.1.06	99.6117	99.6067	99.6092	33.4557	33.4480	33.4867
7.1.07	99.6101	99.6021	99.6081	33.4847	33.5131	33.4889
7.1.08	99.6162	99.6143	99.6093	33.4796	33.4660	33.4484
7.1.09	99.6071	99.6014	99.6093	33.4256	33.4666	33.4549
7.1.10	99.6109	99.5972	99.6090	33.4636	33.5110	33.4284

**Table 12:** Information Entropy analysis of various plain and encrypted test images.

Image	Image Size	Color/Gray	Plain Image			Encrypted Image			Average
			Red	Green	Blue	Red	Green	Blue	
Lena	256 × 256	Gray	7.4429	–	–	7.9974	–	–	7.9974
Peppers	256 × 256	Gray	7.5595	–	–	7.9975	–	–	7.9975
Baboon	256 × 256	Gray	7.2412	–	–	7.9974	–	–	7.9974
Lena	512 × 512	Gray	7.5940	–	–	7.9993	–	–	7.9993
Peppers	512 × 512	Gray	7.5715	–	–	7.9993	–	–	7.9993
Baboon	512 × 512	Gray	7.3579	–	–	7.9992	–	–	7.9992
Lake	512 × 512	Gray	7.4826	–	–	7.9993	–	–	7.9993
Man	512 × 512	Gray	7.5371	–	–	7.9992	–	–	7.9992
Cameraman	512 × 512	Gray	7.0691	–	–	7.9993	–	–	7.9993
Lena (Color)	256 × 256	Color	6.9176	7.5683	7.2353	7.9974	7.9973	7.9973	7.9973
Peppers (Color)	256 × 256	Color	7.0649	7.5084	7.2286	7.9973	7.9973	7.9970	7.9972
4.1.01	256 × 256	Color	6.3807	6.4457	6.4200	7.9971	7.9975	7.9969	7.9972
4.1.02	256 × 256	Color	5.9309	5.9642	6.2499	7.9972	7.9971	7.9974	7.9972
4.1.03	256 × 256	Color	5.7117	5.3738	5.7150	7.9973	7.9971	7.9973	7.9972
4.2.01	512 × 512	Color	6.1265	6.8845	6.9481	7.9993	7.9992	7.9993	7.9993
4.2.03	512 × 512	Color	7.2895	6.3175	6.9294	7.9994	7.9993	7.9994	7.9994
4.2.05	512 × 512	Color	6.2138	6.7990	6.7178	7.9992	7.9992	7.9993	7.9993

**Table 13:** Information entropy comparison of different algorithms (256 × 256).

Images	Ours	Ref. [4]	Ref. [7]	Ref. [31]	Ref. [32]
Lena	7.9974	7.9972	7.9974	7.9975	7.9974
Peppers	7.9975	-	7.9974	7.9965	7.9972

**Table 14:** Information entropy comparison of different algorithms (512 × 512).

Images	Ours	Ref. [37]	Ref. [4]	Ref. [12]	Ref. [13]
Lena	7.9993	7.9992	7.9993	7.9993	-
Peppers	7.9993	7.9993	7.9993	-	7.9992

**Table 15:** Comparison of key space among various algorithms.

Algorithms	Ours	Ref. [31]	Ref. [13]	Ref. [39]
Key space	$2^{732}$	$2^{256}$	$2^{256}$	$2^{472}$

**Table 16:** Time test result of different algorithms.

Image	Encryption Time			Decryption Time		
	Ours	Ref. [4]	Ref. [34]	Ours	Ref. [4]	Ref. [34]
Lena (256 × 256)	0.9782	0.9654	30.1982	0.8775	0.8181	30.3036
Cameraman (256 × 256)	0.9293	0.9144	30.1993	0.8641	0.8167	30.3542
Lena (512 × 512)	3.7066	2.8263	114.7362	3.8762	2.3943	115.9502
Cameraman (512 × 512)	3.7800	2.8040	114.7456	3.6830	2.4038	115.7526
Peppers (512 × 512)	3.7129	2.7894	114.7468	3.6275	2.4205	115.8765
Baboon (512 × 512)	3.7147	2.8805	114.7582	3.6438	2.5077	115.7726

## Data Availability

The original data presented in the study are openly available in the USC-SIPI image database at https://sipi.usc.edu/database/ (accessed on 9 April 2025).
